# A Structural Overview of RNA-Dependent RNA Polymerases from the *Flaviviridae* Family

**DOI:** 10.3390/ijms160612943

**Published:** 2015-06-08

**Authors:** Jiqin Wu, Weichi Liu, Peng Gong

**Affiliations:** 1 Key Laboratory of Special Pathogens and Biosafety, Wuhan Institute of Virology, Chinese Academy of Sciences, No. 44 Xiao Hong Shan, Wuchang District, Wuhan 430071, China; E-Mails: wujiqin2010@163.com (J.W.); liuweichi1990@163.com (W.L.); 2 State Key Laboratory of Virology, Wuhan Institute of Virology, Chinese Academy of Sciences, Wuhan 430071, China; 3 University of Chinese Academy of Sciences, Beijing 100049, China

**Keywords:** *Flaviviridae*, RNA-dependent RNA polymerase, catalytic motif, *de novo* initiation, elongation, *in cis* regulation

## Abstract

RNA-dependent RNA polymerases (RdRPs) from the *Flaviviridae* family are representatives of viral polymerases that carry out RNA synthesis through a *de novo* initiation mechanism. They share a ≈ 600-residue polymerase core that displays a canonical viral RdRP architecture resembling an encircled right hand with palm, fingers, and thumb domains surrounding the active site. Polymerase catalytic motifs A–E in the palm and motifs F/G in the fingers are shared by all viral RdRPs with sequence and/or structural conservations regardless of the mechanism of initiation. Different from RdRPs carrying out primer-dependent initiation, *Flaviviridae* and other *de novo* RdRPs utilize a priming element often integrated in the thumb domain to facilitate primer-independent initiation. Upon the transition to the elongation phase, this priming element needs to undergo currently unresolved conformational rearrangements to accommodate the growth of the template-product RNA duplex. In the genera of *Flavivirus* and *Pestivirus*, the polymerase module in the C-terminal part of the RdRP protein may be regulated *in cis* by the N-terminal region of the same polypeptide. Either being a methyltransferase in *Flavivirus* or a functionally unclarified module in *Pestivirus*, this region could play auxiliary roles for the canonical folding and/or the catalysis of the polymerase, through defined intra-molecular interactions.

## 1. Introduction

The viruses of the *Flaviviridae* family include a large number of important human and animal pathogens with notable members including hepatitis C virus (HCV) from the genus *Hepacivirus*, dengue virus (DENV), Japanese encephalitis virus (JEV), West Nile virus (WNV), and tick-borne encephalitis virus (TBEV) from the genus *Flavivirus*, and bovine viral diarrhea virus (BVDV) and classical swine fever virus (CSFV) from the genus *Pestivirus*. The non-segmented single-stranded RNA genome of these viruses is positive sense and has a typical length of 9.5–12.3 kilo-bases [1,2,3]. The genome contains a large open reading frame (ORF) flanked by 5ʹ and 3ʹ nontranslated regions (NTRs) that usually contain structured element regulating viral genome replication and viral protein translation [4,5,6,7]. The 5ʹ end of the *Flavivirus* genome bears a type 1 cap structure (cap 1) [8], while the genome of other members of *Flaviviridae* is not 5ʹ capped and rather contains an internal ribosome entry site (IRES) in the 5ʹ NTR for cap-independent translation [9,10]. The 3ʹ end of the genome for all *Flaviviridae* members is unexceptionally not poly-adenylated. The single ORF encodes a ≈3000–3900-residue polyprotein that is processed into ≈10–12 structural and non-structural proteins by viral and host proteases [11]. While the structural proteins are key components of viral capsid and envelop, the non-structural proteins all participate in genome replication that occurs in membrane-associated sub-structures derived from and connected to the endoplasmic reticulum [12]. Lying in the heart of the genome replication machinery (also termed the replication complex) is the virally encoded RNA-dependent RNA polymerase (RdRP) that governs the catalysis in synthesizing genome-length RNA. In this review, we present our current understanding of *Flaviviridae* RdRPs primarily from structural perspective with focuses on polymerase catalysis and regulation.

## 2. The Architecture of *Flaviviridae* RdRP Protein and Important Components for Polymerase Catalysis

**Figure 1:**
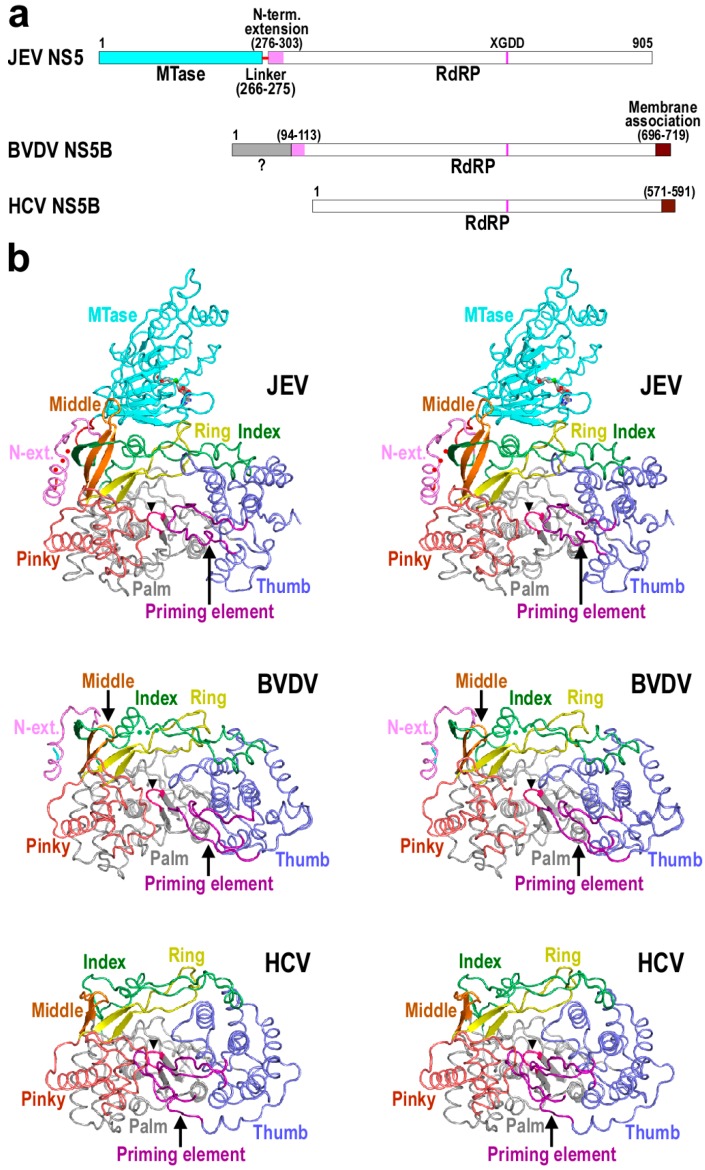
Structural comparison of representative *Flaviviridae* RdRPs. (**a**) A schematic of *Flaviviridae* RdRPs defining functional regions; (**b**) Stereo-pair images of *Flaviviridae* RdRP structures (pdb entries: 4K6M, 1S4F and 1NB4) viewing down into the polymerase active site. The RdRP signature sequence XGDD is shown in magenta (also indicated by a black triangle). Red dots in the JEV structure indicates disordered residues 271–273 in the MTase-RdRP linker and the *S*-adenosyl-l-homocysteine (SAH) bound in the MTase domain is shown as sticks. In the BVDV structure, residues 92–127 from a second molecule are connected to the C-terminal part of the protein by the green dots to present the likely canonical fold. Structures were superimposed using program THESEUS [22].

The viral proteins that carry out RdRP function in family *Flaviviridae* vary in size, with about 600–900 residues encoded. Among these, the nonstructural protein 5 (NS5) of *Flavivirus* is the largest, having a ≈260-residue *S*-adenosyl-l-methionine (SAM)-dependent methyltransferase (MTase) fused to the N-terminus of the polymerase module through a short flexible linker (Figure 1a). Based on consensus folding of the MTase and RdRP in numerous crystal structures [13,14,15,16] and the first full-length NS5 crystal structure in JEV [17], the linker spans about ten residues following a highly consensus GTR sequence in the C-terminus of the MTase. It exhibits tendency to be disordered or to adopt an extended conformation in the majority of structures. As an exception, a helical fold was observed in this region in a recent report of the full-length DENV NS5 structure [18], suggesting its potentials to coordinate domain motions of the regions it is tethering. The interactions and regulations between the MTase and RdRP had remained enigmatic until the first full-length NS5 crystal structure was reported in 2013, starting to unravel the rather complicated nature of the MTase-RdRP interplay [17]. This JEV NS5 structure defines a medium size MTase-RdRP interface with key residues highly conserved in *Flavivirus* and recently proven to be functionally important [19,20]. In contrast to *Flavivirus* NS5, the nonstructural protein 5B (NS5B) of HCV is essentially the polymerase module with a C-terminal 21-residue “membrane anchor” (Figure 1a). The *Pestivirus* NS5B appears to be a “hybrid” with its C-terminal hydrophobic region resembling that of HCV NS5B and its N-terminal 90 residues reminiscent of the *Flavivirus* NS5 MTase (Figure 1a). The *Flavivirus* NS5 and *Pestivirus* NS5B also share a 20–28-residue region first defined in the full-length JEV NS5 structure as “the N-terminal extension” to the core polymerase and recently shown to be non-dispensable to regular polymerase catalysis in JEV NS5 (Figure 1a) [19]. Although sequence similarity of this N-terminal extension is quite low between the two genera, they adopt a similar fold, and if properly presented, were structurally integrated into the core polymerase to form one entirety (Figure 1b) [14,15,17,21].

Viral RdRPs adopt a unique encircled right hand architecture with palm, fingers, and thumb domains surrounding the active site. The fingers domain has been further divided into index, middle, ring, and pinky finger subdomains to better elucidate the RdRP function [17,23] and the encirclement of the active site is achieved through interactions between index finger and thumb (Figure 1b). As a consequence, large-scale rotational conformational changes of the fingers domain typically observed in the nucleotide addition cycle of Pol I family polymerases are not feasible for viral RdRPs [24,25], and they instead utilize small-scale rearrangements in the palm domain to achieve active site closure necessary for the phosphoryl transfer reaction [26], thus providing a structural basis for rational design of active site inhibitors specifically acting on viral RdRPs. With respect to the initiation mechanism of RNA synthesis, viral RdRPs can be classified into two major classes. The *Flaviviridae* core polymerase represents *de novo* RdRPs that utilize two initiating NTPs to form the first phosphodiester bond of the product strand [27,28]. The other class is represented by *Picornaviridae* (e.g., poliovirus, or PV) RdRPs that take advantage of a primer (a virally encoded peptide in PV) in the early stages of RNA synthesis [29]. One primary structural difference between *de novo* and primer-dependent RdRPs is that the former often have a priming element integrated into their thumb domain. This priming element penetrates into the active site from the upstream direction (Figure 1b), allows only the 3ʹ portion of the template strand to approach the active site from the downstream for initiating NTP binding, and facilitates the first few steps of RNA synthesis [28].

**Figure 2:**
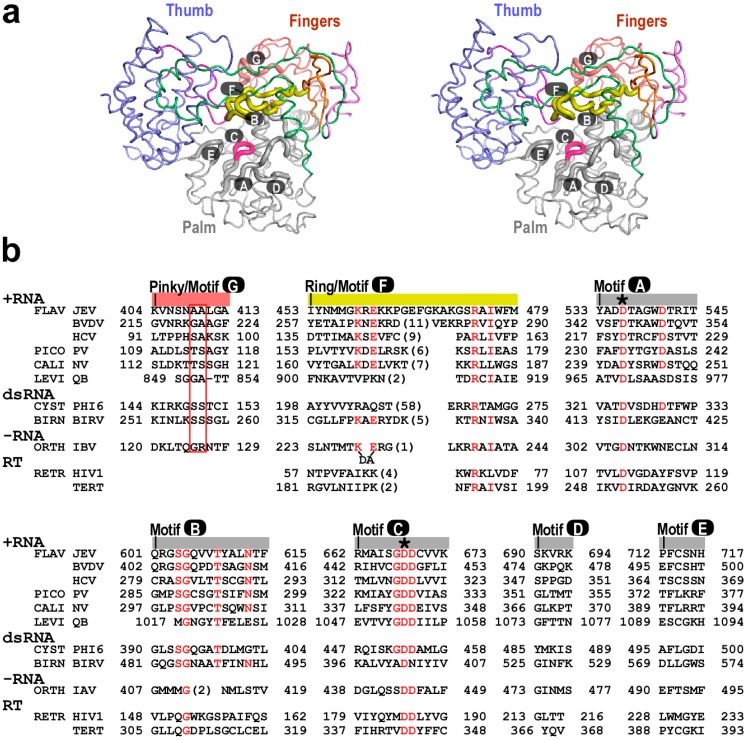
Catalytic motifs of *Flaviviridae* RdRPs. (**a**) Stereo-pair images of spatial organization of the JEV RdRP catalytic motifs A–G (pdb entry: 4K6M). Seven RdRP motifs are shown as thick noodles. The color-coding is as in Figure 1b; (**b**) A structure-based sequence alignment depicting the conservation of RdRP motifs (pdb entries: 4K6M, 1S4F, 1NB4, 3OL6, 3BSO, 3AVT [36], 1HI0, 2PGG [37], 4WRT [38], 1RTD [39], and 3DU6 [40]). Three RdRPs from other positive-strand RNA viruses (PICO/PV: *Picornaviridae*/poliovirus; CALI/NV: *Caliciviridae*/norovirus; LEVI/QB: *Leviviridae*/bacteriophage Qβ), two from double-stranded RNA viruses (CYST/PHI6: *Cystoviridae*/bacteriophage ϕ6; BIRN/BIRV: *Birnaviridae*/birnavirus), one from negative-stranded RNA viruses (ORTH/IBV: *Orthomyxoviridae*/influenza virus B), and two reverse transcriptases (RTs) (RETR/HIV1: *Retroviridae*/human immunodeficiency virus 1; TERT: Telomerase RT) were chosen as representatives for alignment and/or comparison. Only the structurally conserved segment of motif D is included in this alignment. Some important RdRP consensus residues are highlighted in red. Two motif G residues interacting with the +1/+2 junction of the RNA template are indicated by a red box. The two universal aspartic acid residues are indicated by asterisks. Numbers in parenthesis indicate the number of residues not shown. The alignment of IBV polymerase motif G is of lower confidence due to a lower level of structural similarity to motif G in other structures.

For all viral RdRPs, the palm domain harbors polymerase motifs A–E with the most conserved feature embedded in motifs A–C [30,31]. Motifs A and C each contains an aspartic acid residue that is universally conserved for all single-subunit processive nucleic acid polymerases (Figure 2b), playing central roles in the two-metal ion catalytic mechanism [17,32]. Motif B includes a highly conserved serine (JEV NS5 residue 604 or equivalent) known to play key roles in recognizing the 2ʹ-hydroxyl group of the NTP ribose [26,33]. It also contains a glycine residue (JEV NS5 residue 605 or equivalent) that is conserved in all RNA-dependent polymerases. This glycine not only provides flexibility for the Ser-Gly peptide bond flip to coordinate the side-chain rotamer change of the adjacent serine in active site closure [26], but also may play a key role in polymerase translocation. In a recent study, a loop region (JEV NS5 residues 603–609 or equivalent) of PV RdRP centering around this glycine was shown to adopt two distinct backbone conformations and was proposed to mediate the movement of the template-product duplex toward the upstream in the post-catalysis translocation event [34]. Motifs D and E are less conserved in sequence. Structurally, motif D is associated with motif A and they undergo coordinated conformational changes during the closure and reopening of the active site [26]. A motif D lysine residue (K359 in PV RdRP) that is conserved in primer-dependent RdRPs has been proposed to play critical roles in catalysis [35]. However, the region spanning this residue does not have equivalents in *de novo* RdRPs, either by sequence or by structural homology. Motif E folds with motif C, and primarily interacts with the backbone of the −2 and −3 positions (*i.e.*, the 2nd and the 3rd nucleotide upstream of the active site) of the product RNA.

Motifs F and G reside in the fingers domain, and both play critical roles for polymerase function. Motif F is common for all RNA-dependent polymerases and its functions are yet to be clarified [41]. When properly folded, it forms the roof of the NTP entry channel and adopts an antiparallel β-type structure with key residues (JEV NS5 K459 and E461 or equivalent) in the N-terminal half approaching the nascent base pair through the hoogsteen face and those (JEV NS5 R474 and I476 or equivalent) in the C-terminal half interacting with the base pair plane and the NTP triphosphate moiety [26,28,42]. The N-terminal half of motif F was also proposed to mediate stem-loop A (SLA)-promoted RNA synthesis in DENV, suggesting possible different roles of motif F in different stages of RNA synthesis [43]. Note that not all RdRPs motifs exhibit high level of sequence conservation, even if they were originally identified based on sequence homology among a collection of viral species [30]. Based on known RdRP-RNA structures [26,44,45,46], Motif G runs approximately parallel to the template strand with two residues (JEV NS5 A409-A410 or equivalent) running vertically to the +1/+2 backbone kink of the RNA template. Although sequence conservation is quite low across the representative viral species for motif G, in particular, between primer-dependent and *de novo* RdRPs, its critical location implies potential key roles in binding and/or translocating the RNA template strand.

## 3. Structural Basis for Polymerase Catalysis

**Figure 3:**
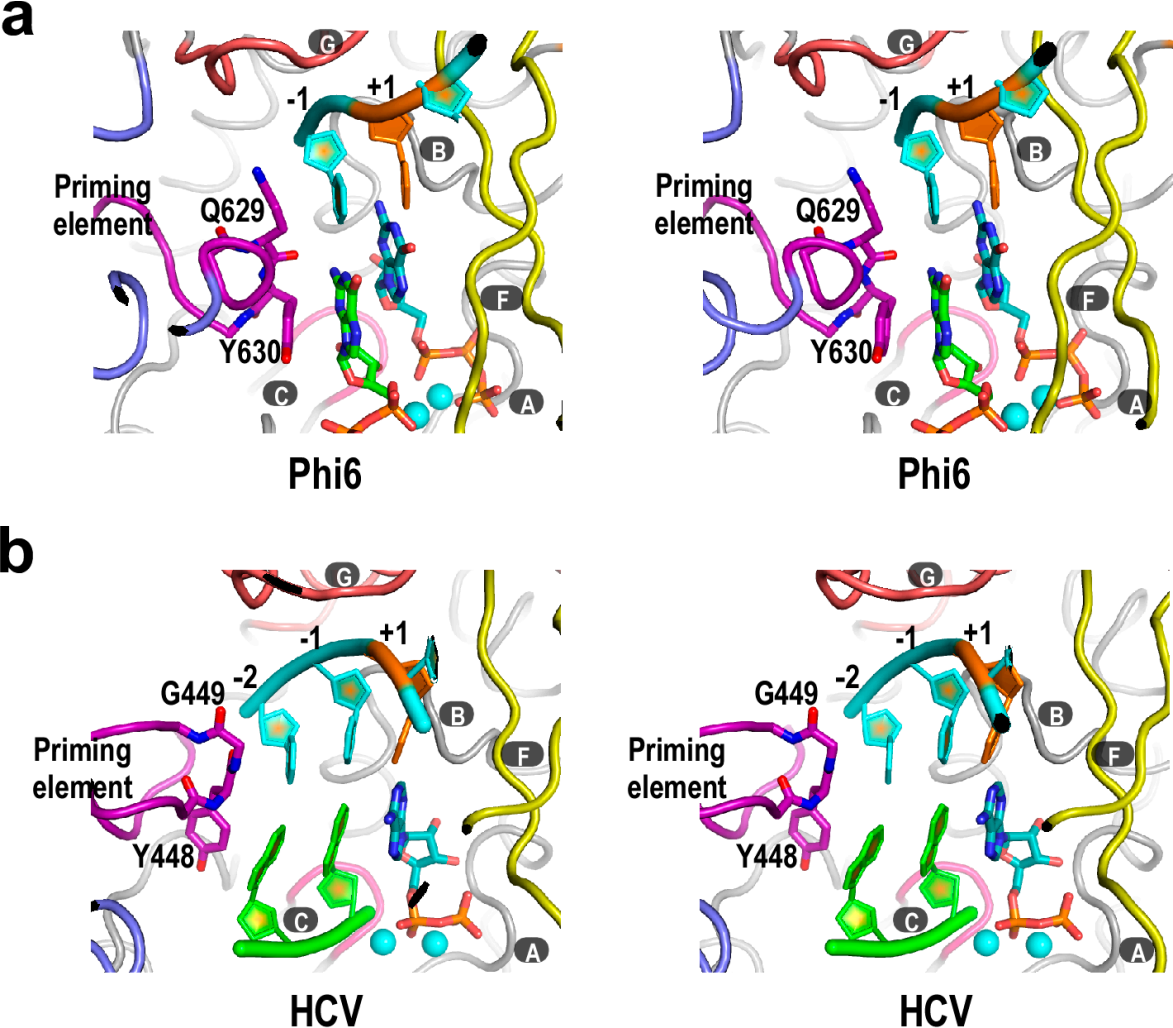
The crystal structures of *de novo* RdRP initiation complex (IC**)**. (**a**) Bacteriophage ϕ6 polymerase IC structure (pdb entry: 1HI0). Template RNA is in cyan. Initiation NTPs and residues Q629 and Y630 are shown as sticks, and magnesium ions are shown as cyan spheres; (**b**) HCV NS5B IC structure (pdb entry: 4WTJ). Template RNA is in cyan and dinucleotide primer is in green. ADP and residues Y448 and G449 are shown as sticks, and manganese ions are shown as cyan spheres. Structure superimpositions were carried out using motif C residues and the least-square method. For both panels, the +1 templating nucleotide is shown in orange. Capital letters with dark grey background indicate RdRP catalytic motifs.

Analogous to DNA-dependent RNA polymerases [47,48,49], an initiation complex (IC) of *Flaviviridae* RdRP is unstable, and tends to release short RNA products and re-initiate [27]. However, precise and relatively efficient initiation can be achieved with the help of the priming element. Note that the priming element is structurally quite diverse across different viruses (Figure 1b). It could comprise solely an extended loop structure, as in *Flavivirus* NS5 [14,15,17], or two peptide segments that contain a β-hairpin structure, as in HCV NS5B [50], or even a helical module, as in bacteriophage ϕ6 polymerase [28]. Crystal structures of the *de novo* RdRP IC from ϕ6 and HCV have been reported, providing the structural basis for how the early stages of RNA synthesis is generally achieved with the assistance of the priming element (Figure 3). In the ϕ6 polymerase IC structure, residues Q629 and Y630 of the priming element were placed at the immediate upstream position of the initiation site, stacking onto the −1 templating nucleotide and the priming NTP, respectively (Figure 3a) [28]. Such an arrangement likely ensures terminal initiation for faithful replication, and may also stabilize the priming NTP for catalysis. In the recently reported set of HCV NS5B IC structures, different dinucleotides were used as short primers to mimic the situation in *de novo* initiation [42]. Similar to the ϕ6 polymerase IC, priming element residues Y448 and G449 interact with the upstream end (now position −2 *vs.* −1 in ϕ6 structure) of the template and product strands, respectively (Figure 3b). Note that the priming element could contribute to efficient initiation, or precise start site selection, or both. In an earlier report, when the β-hairpin of the HCV NS5B priming element was truncated for eight residues (residues 444–447 and 450–453 were removed, and residues 448–449 were replace by two glycines to accommodate the truncation), the *de novo* synthesis activity was not much affected, but this mutant NS5B lost the specificity of terminal initiation and could initiate from internal sites of a template [51]. In a very recent report, mutation of hydrophobic and charged residues within the priming element of HCV NS5B resulted in a substantial decrease of *de* *novo* initiation activities [52]. These data indicate that the priming element does play essential roles in *de novo* initiation, although the mechanistic details remain to be further clarified. Similar initiation mechanisms may be taken by RdRPs from *Flavivirus* and *Pestivirus* genera, and several residues within the priming element of DENV NS5 has been proposed to contribute to the initiation-related processes [53]. However, how the initiation platform is set up in these RdRPs still awaits clarification from relevant IC structures. In contrast to *de novo* viral RdRPs that take advantage of the priming element at initiation, primer-dependent viral RdRPs represented by PV 3D^pol^ utilize a protein primer (termed VPg for “viral protein genome-linked”) or its uridylylated forms to initiate RNA synthesis [54,55]. To date, RdRP-VPg complex crystal structures have been reported in foot-and-mouth disease virus (FMDV), coxsackievirus B3 (CV B3), and enterovirus 71 (EV71), each displaying a distinct mode of interactions between the polymerase and VPg [56,57,58]. However, how VPg or uridylylated VPg is utilized in the template-directed polymerase initiation remains elusive, as none of the three polymerase-VPg structures includes an RNA template or provides clear clues for how such an IC is spatially arranged.

The ϕ6 and HCV polymerase IC structures could represent the situation in the first and second nucleotide addition cycles of the initiation process, respectively. From these one could deduce the possible outcome as the template-product RNA duplex grows to a length of about 7–8 base pairs and occupies the entire front channel of the polymerase, a status observed in multiple *Picornaviridae* RdRP elongation complex (EC) crystal structures [26,44]. Presumably, the priming element would withdraw from the active site to accommodate the growth of RNA duplex, leading to a substantial but currently unresolved conformational change in the thumb domain. Whether the priming element adopts a certain fold in an RdRP EC, and whether it establishes new interactions with other regions of the EC or contributes to the processivity of the EC, are yet to be clarified. To date, solving a crystal structure of an EC with intact priming element remains a challenge [42,59], and if fulfilled, would be critical for answering these questions.

Among other basic aspects of RdRP catalysis, the details of elongation catalytic cycle are largely clarified in PV 3D^pol^, a representative of primer-dependent RdRP [26]. In this multi-step catalytic cycle, a pre-positioned +1 templating nucleotide directs the incoming NTP to bind in a site very close to its catalytic position. Next, the 2ʹ and 3ʹ hydroxyl groups of the NTP ribose likely trigger the active site closure featuring the movement of motifs A and D to allow a critical aspartic acid residue in motif A (JEV NS5 residue 536 or equivalent) to participate in the coordination of two catalytic magnesium ions. This closure of the active site leads to the phosphoryl transfer reaction, and is generally accepted as one of the slow steps in the catalytic cycle. Important post-catalysis events include the release of the pyrophosphate byproduct, the re-opening of the active site, and polymerase translocation to realign the active site to the next register on the template RNA. It is worth mentioning that the mechanism of translocation of viral RdRPs may be the most important part of the cycle that remains elusive [26]. Translocation of the single subunit Pol I family polymerases is coupled with active site reopening. In such a reopening process, the “O helix” in the fingers domain rotates significantly, and utilizes an aromatic side chain to push the nascent base pair toward the upstream [24,25]. As mentioned above, viral RdRPs close and reopen the active site primarily through the movement of motifs A and D in the palm, and there is no O helix equivalent in these proteins. Therefore, a different translocation mechanism has to be taken by viral RdRPs. Structurally, motif B intimately interacting with the nascent base pair on one side of the template strand and motif G that holds the other side of the template strand and makes close contact with the backbone of the template +1/+2 kink are the best candidates to participate in translocation [17,34]. However, more biochemical evidences as well as structural biology efforts in capturing possible low energy translocation intermediates are necessary to advance our understanding of this missing link in the RdRP catalytic cycle.

## 4. *Flaviviridae* RdRP Regulation in *Cis*

**Figure 4:**
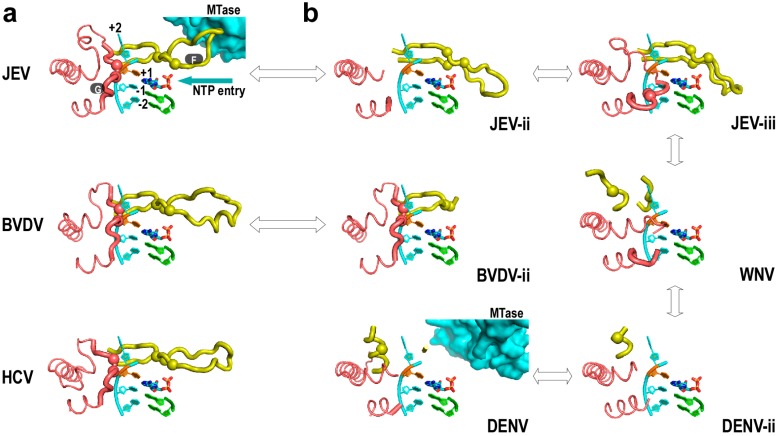
Conformational heterogeneity of *Flaviviridae* polymerase structure may be related to *in cis* regulations. (**a**) Representative *Flaviviridae* polymerase structure adopting the canonical conformations (pdb entries: JEV/4K6M, BVDV/1S4F, HCV/1NB4); (**b**) Disorder and/or alternative folding of motifs F and G observed in *Flaviviridae* polymerases (pdb entries: JEV-ii/4MTP [60], JEV-iii/4HDG, WNV/2HFZ, DENV/4V0Q, DENV-ii/2J7U, BVDV-ii/2CJQ). Motifs F and G are shown as thick noodles and the pinky finger residues flanking motif G are shown as thin noodles. Note that in the apo JEV RdRP structure (JEV-ii) and GTP-bound JEV RdRP structure, the NTP entry channel is blocked by the non-canonically folded motif F. Color coding is as in Figure 1b and Figure 3b. A 4 nt RNA template, a dinucleotide primer, and an ADP molecule taken from an HCV IC structure (pdb entry: 4WTJ) were modeled into all structures for comparison. The α-carbons of JEV NS5 residues 409–410 in motif G and 459, 461, 474 in motif F and their equivalents in other polymerases are shown as spheres to help distinguish canonical and alternative folding of these two motifs. Double arrows are used to connect polymerases from the same viral species or from the same genus. The MTase in the full-length *Flavivirus* NS5 structures are shown as surface representations. Structure superimpositions were carried out as in Figure 1b.

In contrast to HCV NS5B, JEV NS5 and BVDV NS5B each has an N-terminal region that may provide *in cis* regulation of the core polymerase. This difference is reflected in the crystal structures of these RdRPs to some extent (Figure 4). The structure of HCV NS5B exhibits a canonical RdRP fold (Figure 4a, bottom panel) with all seven catalytic motifs properly arranged around the active site [50]. By contrast, the crystal structures of the *Flavivirus* NS5 in the absence of the N-terminal MTase consistently exhibit fingers domain disorder or non-canonical folding, particularly for motifs F and G (Figure 4b, JEV-ii, JEV-iii, WNV, and DENV-ii structures) [14,15,60]. It was until the report of the first full-length NS5 crystal structure that the intact and canonically folded *Flavivirus* polymerase was observed (Figure 4a, top panel). In this full-length JEV NS5 structure, motif F participates in an intra-molecular interface between MTase and polymerase and contributes Phe467 to the formation of a six-residue hydrophobic network [17]. These hydrophobic residues are highly conserved in genus *Flavivirus*, implying the functional significance of the interface. When polar or charged residue mutations were introduced into these sites, virus replication levels were significantly affected [20]. Furthermore, the introduction of negatively charged Asp mutation into these sites could affect *in vitro* polymerase activities at both initiation and elongation [19]. Taken together, the MTase-polymerase interface observed in the full-length JEV NS5 structure play auxiliary roles to fingers domain folding and polymerase catalysis. In DENV NS5 studies, the removal of the MTase domain and/or further removal of the linker residues were shown to alter polymerase activities [61,62]. Very recently, a full-length DENV NS5 crystal structure was reported, and a different interface between MTase and polymerase was observed with less hydrophobic features [18]. In such a structure, motif F no longer participates in the interface and both motif F and motif G are largely disordered (Figure 4b, DENV structure) as with those MTase-less NS5 structures. As a natural fusion of MTase and polymerase, the full-length *Flavivirus* NS5 may have the MTase interact with the polymerase in different ways. However, the conformational state observed in the full-length JEV NS5 crystal structure with canonically folded motifs F and G may be regarded as the ground state for polymerase catalysis, as the highly conserved K/E/R residues in motif F (JEV NS5 residues 459, 461, 474 or equivalent) and the structurally critical residue pair in motif G (JEV NS5 409–410 or equivalent) are in-line for NTP/+1 templating nucleotide interactions and for template RNA binding, respectively (Figure 2b and Figure 4a). Although the binding of NTP or an inhibitor molecule could affect the folding of motif F in JEV RdRP structures [60] or motif G in a DENV RdRP structure [63], respectively, the conformations of the NTP/inhibitor-induced regions apparently deviate from the canonical folds (see Figure 4b and JEV-iii for the conformation of the JEV RdRP-GTP structure).

The N-terminal region of *Pestivirus* NS5B contains ≈90 residues, and its function remains elusive. It is less likely to form a relatively independent enzyme module due to its limit in size, but could regulate the function of polymerase through intra-molecular interactions as observed in *Flavivirus* NS5. Two basic conformational states have been observed in reported BVDV NS5B crystal structures obtained using N-terminal truncated proteins. In one conformational state, the majority of the polymerase (residue 134 and beyond) adopts the canonical fold (Figure 4a, middle panel). However, residues 92–133 exist in a domain swapped mode and fold with another NS5B molecule [64]. The other conformational state was obtained using an NS5B carrying an Asn438 duplication [21]. In such a structure, residues 92–133 now folds with the rest of the same polypeptide chain. Interestingly, motif F and index finger in the Asn438 duplication structure were partially disordered (Figure 4b, BVDV-ii structure, index finger not shown), reminiscent of what is observed in *Flavivirus* polymerase structure in the absence of the N-terminal MTase. Therefore, it is also possible that the N-terminal region of *Pestivirus* NS5B could contribute to the proper folding and/or the catalytic regulation of the polymerase through yet unidentified intra-molecular interactions. Indeed, deletions within the N-terminal region could reduce or abolish *de novo* RNA synthesis activities in BVDV and CSFV systems [65,66].

## 5. Perspectives

As ideal systems to study the function of *de novo* viral polymerases, *Flaviviridae* RdRPs share a polymerase module that combines common RdRP motifs A through G with a priming element essential for primer-independent initiation. Major challenges remain in elucidating the mechanism of polymerase translocation on the RNA template and the conformational rearrangements primarily involving the priming element in transition from polymerase initiation to elongation. Within the two motifs most probable in participating the translocation process, motif B is shared by viral RdRPs and reverse transcriptases (RTs) and motif G is RdRP specific. Whether or not the primary translocation mechanism is shared by RdRPs and RTs, it probably differs from what is taken by the Pol I family polymerases based on structural divergences. In practice, the mystery of the priming element conformational changes can be uncovered in any *de novo* viral RdRPs systems including those from *Cystoviridae*. With the low level of sequence conservation of the priming element considered, the details of the rearrangement during the transition process could be very different. However, the general strategy to complete these conformational changes is likely to be similar.

The *in cis* regulation on some of the *Flaviviridae* RdRPs by their N-terminal domain has brought in interactions and functions beyond the fundamental processes of RdRP catalysis. In *Flavivirus* NS5, crystal structures of the full-length protein have shed light on how the regulation could take place, and also provide valuable clues to investigate the function of NS5 beyond polymerase catalysis. *Pestivirus* NS5B still awaits a high-resolution full-length structure to reveal the true face of the N-terminal domain and its relationship with the polymerase module.
